# Analgesia by intrathecal delta-9-tetrahydrocannabinol is dependent on Cav3.2 calcium channels

**DOI:** 10.1186/s13041-023-01036-8

**Published:** 2023-05-25

**Authors:** Vinicius de Maria Gadotti, Flavia Tasmin Techera Antunes, Gerald W. Zamponi

**Affiliations:** 1 Department of Clinical Neurosciences, University of Calgary, Calgary, AB Canada; 2 Alberta Children’s Hospital Research Institute, University of Calgary, Calgary, AB Canada; 3 Hotchkiss Brain Institute, University of Calgary, Calgary, AB Canada; 4 Cumming School of Medicine, University of Calgary, Calgary, AB Canada

**Keywords:** Δ^9^-THC, Cannabinoid receptors, Cav3.2 channel, Analgesia, Pain

## Abstract

Delta-9-tetrahydrocannabinol (Δ^9^-THC) is known to produce systemic analgesia that involves CB_1_ and CB_2_ cannabinoid receptors. However, there is compelling evidence that Δ^9^-THC can potently inhibit Cav3.2T-type calcium channels which are highly expressed in dorsal root ganglion neurons and in the dorsal horn of the spinal cord. Here, we investigated whether spinal analgesia produced by Δ^9^-THC involves Cav3.2 channels vis a vis cannabinoid receptors. We show that spinally delivered Δ^9^-THC produced dose-dependent and long-lasting mechanical anti-hyperalgesia in neuropathic mice, and showed potent analgesic effects in models of inflammatory pain induced by formalin or Complete Freund’s Adjuvant (CFA) injection into the hind paw, with the latter showing no overt sex differences. The Δ^9^-THC mediated reversal of thermal hyperalgesia in the CFA model was abolished in Cav3.2 null mice, but was unaltered in CB_1_ and CB_2_ null animals. Hence, the analgesic effects of spinally delivered Δ^9^-THC are due to an action on T-type calcium channels, rather than activation of spinal cannabinoid receptors.

**Supplementary Information:**

The online version contains supplementary material available at 10.1186/s13041-023-01036-8.

T-type Ca^2^^+^ channels are known to be important regulators of pain transmission in primary afferent sensory neurons and the spinal cord [1]. Among the three isoforms of T-type Ca^2^^+^ channels that are expressed in the mammalian genome, the Cav3.2 channel isoform appears to be the predominant T-type channel subtype involved in this process [2]. It is expressed in a subpopulation of primary afferent fibers and the spinal dorsal horn [3], and its expression is enhanced in these tissues in a wide range of chronic pain conditions in rodents [1]. Consequently, systemic or intrathecal delivery of T-type channel inhibitors mediates analgesia (for review see [1, 4]. T-type channels can be inhibited by different types of endocannabinoids [5], terpenes [6] and phytocannabinoids such as cannabidiol and Δ^9^-THC [7, 8]. In particular, Δ^9^-THC mediates strongly state dependent inhibition of Cav3.2 channels with a preference for binding to inactivated channels [7, 8]. It is known that spinally delivered Δ^9^-THC inhibits mechanical and cold allodynia in models of neuropathic pain [9], and analgesia exerted by Δ^9^-THC delivered to the brain involves modulation of both CB_1_ and CB_2_ receptors [10]. However, it is unclear whether the spinal actions of Δ^9^-THC involve T-type channels, cannabinoid receptors, or a combination thereof. Thus, the present study was designed to investigate contributions of spinal CB receptor subtypes and Cav3.2 channels on the antihyperalgesic effect of spinally delivered Δ^9^-THC. All experiments were carried out with approval of an animal protocol by the Institutional Animal Care and Use Committee, and all efforts were made to minimize animal suffering according to the policies and recommendations of the International Association for the Study of Pain. Δ^9^-THC was delivered by intrathecal injection as described before [6, 8] into male and female C57BL/6J (wild-type), or male CB1 null, CB2 null, or Cav3.2 null mice (20 − 25 g, 8–10 weeks; Jackson Laboratories). We first assessed the analgesic action of spinally delivered Δ^9^-THC in the acute nociceptive (phase 1) and inflammatory pain (phase 2) phases of a standard formalin test [11]. Intrathecally delivered Δ^9^-THC, 20 min before testing, significantly and dose-dependently reduced the duration of nocifensive responses in the first (Fig. 1a) and second (Fig. 1b) phases of the formalin test. Next, we verified whether spinal Δ^9^-THC was also able to inhibit mechanical hyperalgesia caused by chronic neuropathy 21 days after partial sciatic nerve injury which was performed as described by us before [11]. Mechanical hyperalgesia was measured using a Dynamic Plantar Aesthesiometer (Ugo Basile, Varese, Italy). When compared to the neuropathic control group, treatment of mice with Δ^9^-THC (10.0 µg/i.t.), but not vehicle (PBS, 10 µl/i.t.) produced marked anti-hyperalgesia when evaluated 45 min after treatment (Fig. 1c). These data show that Δ^9^-THC mediates robust analgesia in wild type mice. Next, we investigated the effect of Δ^9^-THC in a model of persistent inflammatory pain. 20 μl of Complete Freund's Adjuvant (CFA) were given intraplantarly (i.pl.) in the ventral surface of the right hindpaw, whereas sham groups received 20 μl of PBS. Thermal hyperalgesia was examined by measuring the latency to withdrawal of ipsilateral hind paws in response to a focused beam of radiant heat (IR = 30) using a plantar test apparatus (UgoBasile, Varese, Italy). Two days after CFA injection, intrathecal treatment with Δ^9^-THC (10 µg/i.t) but not with vehicle (10 µl/i.t.) resulted in anti-hyperalgesia that remained significant up to 3 h (Fig. 1d). Δ^9^-THC was also effective in increasing paw withdrawal latencies when delivered to female mice (tested 45 min after its spinal delivery, Fig. 1e). To determine whether the analgesic effects observed for spinally delivered Δ^9^-THC were mediated by cannabinoid CB_1_ or CB_2_ receptors, we repeated the CFA model using male CB_1_ (Fig. 1f) and male CB_2_ (Fig. 1g) null mice in comparison with male wild-type mice that were simultaneously tested. For this purpose, mice were injected intrathecally with either vehicle (control) or Δ^9^-THC (10.0 µg/i.t.) and tested 45 min later. Similar to wild type animals, Δ^9^-THC produced significant analgesic effects, indicating that neither of these two receptors are essential for the observed analgesia even though this compound is an agonist of both receptor types [12].

**Fig. 1:**
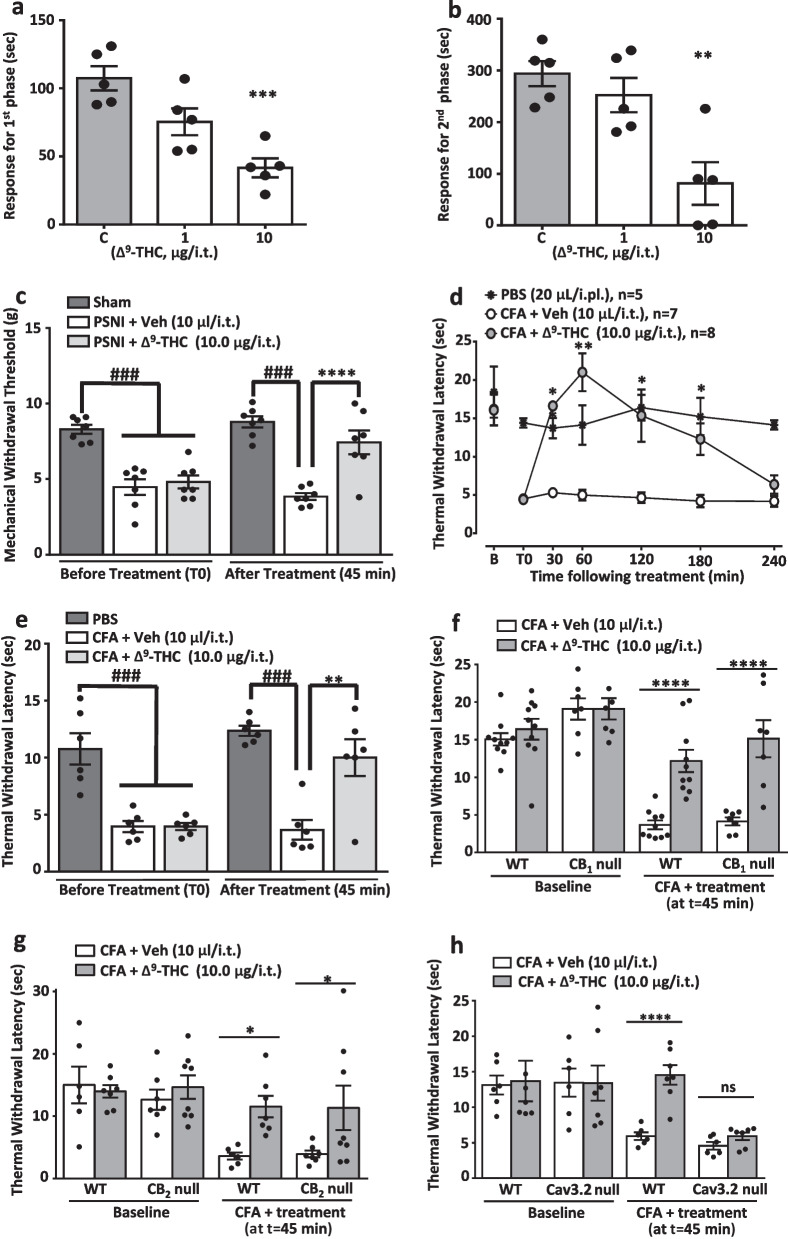
Δ^9^-THC produces spinal analgesia in mice that is Cav3.2 channel-dependent. Dose response action of Δ^9^-THC (delivered 20 min before formalin) in the **a** first and **b** second phases of the formalin test. Each bar represents the mean of 5 animals, error bars denote S.E.M. Data are representative of 2 independent sets of experiments. Statistical analyses were performed by two-way ANOVA followed by Tukey's test. Asterisks denote a significant difference, **P < 0.01 and ***P < 0.001 when compared with the control groups. **c** Mechanical threshold of PSNI mice 45 min after treatment with Δ^9^-THC (10 ug/i.t.). Bars represent the mean of 7 animals, error bars denote S.E.M. Data are representative of 2 independent sets of experiments. Two-way ANOVA followed by Tukey's test revealed significance, ###P < 0.001 and ****P < 0.0001 when compared with the control groups. **d** Time-course of the effect of Δ^9^-THC (10 μg/i.t.) on thermal withdrawal latencies of CFA-injected male mice. **e** Effect on CFA-treated female mice when evaluated 45 min following treatment. In **d** and **e**, error bars are S.E.M. Data are representative of 2 independent sets of experiments Two-way ANOVA followed by Tukey's test revealed statistical differences, *P < 0.05 **P < 0.01 or ***P < 0.001 when the CFA + treated group is compared with the CFA + vehicle control group, and ^###^P < 0.001 when the PBS group is compared with the  control groups. **f**, **g** Comparison of the effect of 10 μg/i.t. Δ^9^-THC on CFA-injected wild type and **f** CB_1_**, g** CB_2_, and **h** Cav3.2 knockout mice. Each bar represents the mean of 6–10 mice, error bars are S.E.M. Data are representative of 2 independent sets of experiments. Two-way ANOVA followed by Tukey's test revealed statistical differences, *P < 0.05 or ****P < 0.0001 when compared with the control group

We then tested the analgesic effect of spinal Δ^9^-THC in Cav3.2 null mice. These mice develop CFA-induced hypersensitivity despite the absence of Cav3.2 channels [11], most likely due to compensatory mechanisms that are not fully understood. As shown in Fig. 1h, Δ^9^-THC lost its analgesic effects when delivered to Cav3.2 null mice, indicating that the key biological target for spinally delivered Δ^9^-THC are T-type channels.

There is considerable evidence that CB_1_ receptor activation mediates analgesia [13], however there are also reports that the analgesic activity of Δ^9^-THC is lost in CB receptor null mice [10, 14]. We do not challenge a possible involvement of these receptors when Δ^9^-THC is delivered systemically. Our focus was to specifically isolate a spinal effect, and this can be cleanly accomplished by the intrathecal route of delivery used in our study (Additional file 1: Fig S1). What we do not know is the overall contribution of the spinal action to the overall analgesic properties of Δ^9^-THC. We attempted testing the effect of systemically delivered Δ^9^-THC in Cav3.2 null mice, however, we found that these mice became lethargic, thus confounding the types of pain behavioral measurements that we typically perform. Finally, our laboratory has previously reported that the analgesic effect of intrathecally delivered mixed CB receptor/Cav3.2 ligands are abolished in Cav3.2 null mice, but they retain activity upon blocking CB_1_ receptors with AM-281 [15]. Interestingly, inhibition of CB_2_ receptors with AM-630 did attenuate the analgesic effects of these compounds and we concluded that although CB_2_ receptors may be involved in their actions, this may be due to CB_2_ receptor modulation of Cav3.2 channel activity. Hence, we cannot rule out the possibility that Δ^9^-THC might activate spinal CB_2_ receptors which may in turn inhibit Cav3.2 in addition to the direct inhibitory actions of Δ^9^-THC on these channels. We note that CB_1_ receptors do not functionally inhibit Cav3.2 in heterologous systems [8] but we could at that time not explore such coupling for CB_2_ receptors for technical reasons. Nonetheless, even if CB_2_ receptors augment direct inhibition of Cav3.2 channels, our results clearly implicate Cav3.2 channels as an essential target of Δ^9^-THC in the actions of spinal Δ^9^-THC as an analgesic, whereas CB receptors are not required.

## Supplementary Information

**Additional file 1:** **Figure S1:** Graphical representation of the primary afferent pain pathway. Intrathecal injection of Δ9-THC induces analgesia in mice lacking either the CB1 or the CB2 receptor, but not in Cav3.2 null mice.

## Data Availability

All data generated or analysed during this study are included in this published article.

## Abbreviations

Δ^9^-THC: Delta-9-tetrahydrocannabinol
CB: Cannabinoid
CFA: Complete Freund’s adjuvant
PSNI: Partial sciatic nerve injury
i.t.: Intrathecal
