# Supplementary material for: Analgesia by intrathecal delta-9-tetrahydrocannabinol is dependent on Cav3.2 calcium channels
Source: Mol Brain. 2023 May 25;16:47. doi: 10.1186/s13041-023-01036-8 (PMC10210369; doi:10.1186/s13041-023-01036-8)
Supplement: Supplementary file 1 — Additional file 1: Figure S1: Graphical representation of the primary afferent pain pathway. Intrathecal injection of Δ9-THC induces analgesia in mice lacking either the CB1 or the CB2 receptor, but not in Cav3.2 null mice. [file 13041_2023_1036_MOESM1_ESM.pdf]

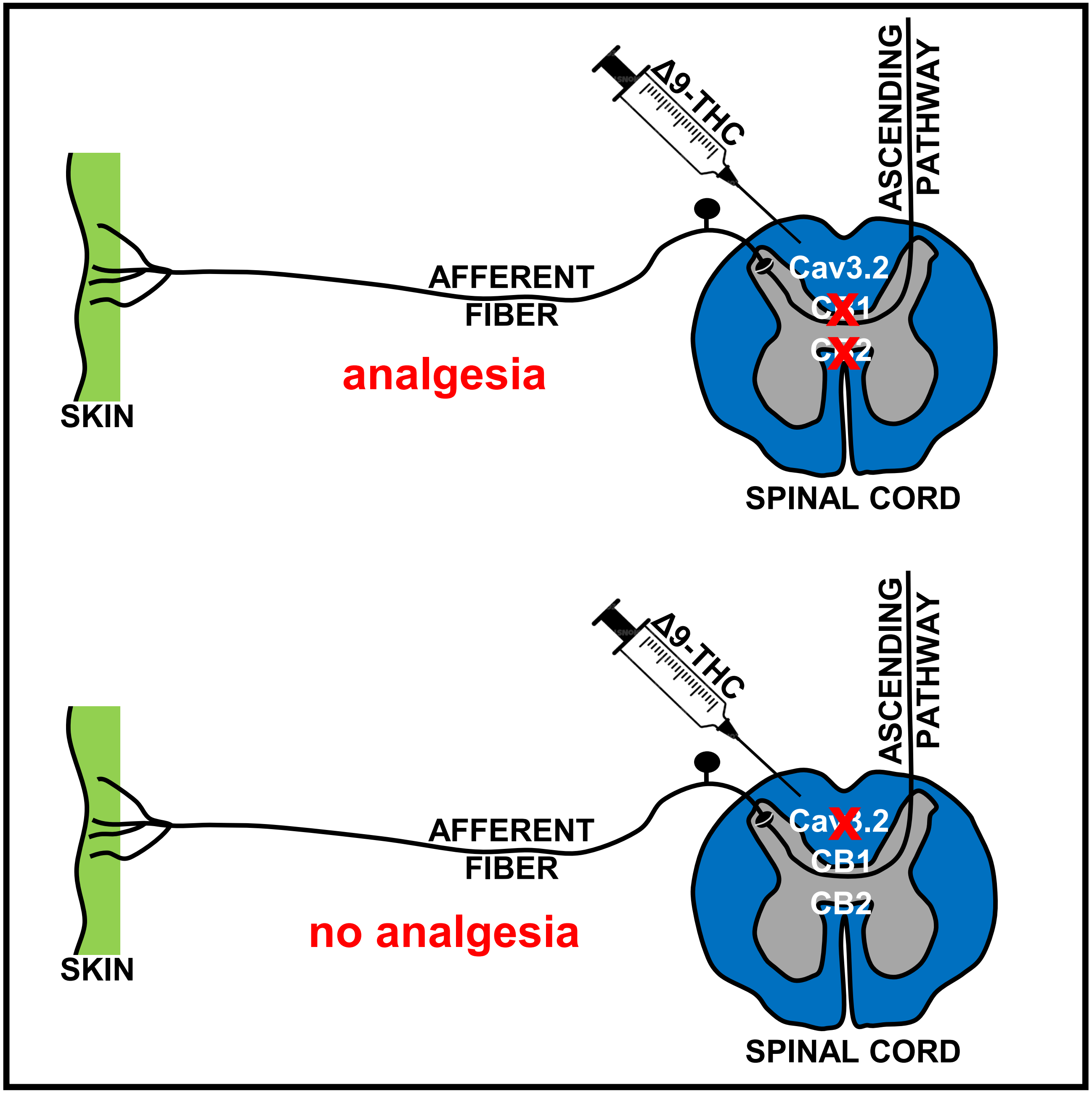

Figure S1: Graphical representation of the primary afferent pain pathway. Intrathecal injection of Δ9-THC induces analgesia in mice lacking either the CB1 or the CB2 receptor, but not in Cav3.2 null mice.
